# Impact of hospital educational environment and occupational stress on burnout among Greek medical residents

**DOI:** 10.1186/s13104-019-4326-9

**Published:** 2019-05-22

**Authors:** Efstathios Papaefstathiou, Andreas Tsounis, Eirini Papaefstathiou, Maria Malliarou, Theodoros Sergentanis, Pavlos Sarafis

**Affiliations:** 1 Faculty of Social Sciences, Hellenic Open University, Patras, Greece; 2 School of Psychology, Aristotle University of Thessaloniki, Thessaloniki, Greece; 3 School of Medicine, National and Kapodistrian University of Athens, Athens, Greece; 4 Department of Nursing, Technological Educational Institute of Thessaly School of Health Sciences, Larissa, Greece; 5 Department of Nursing, School of Health Sciences, Cyprus University of Technology, 15, Vragadinou Str, 3041 Limassol, Cyprus

**Keywords:** Burnout, Stress, Hospital, Quality of education, Residents

## Abstract

**Objective:**

A number of risk and protective factors have been described on the development of burnout syndrome amongst medical residents. The current study aims to investigate the impact of hospital educational environment and occupational stress on trainee doctors burnout. A cross-sectional study among 269 medical residents was conducted. Greek version of Postgraduate Hospital Educational Environment Measure (PHEEM-G) for the assessment of their educational environment, Greek Version of Job Stress Measure (JSM-G) for the stress assessment and Copenhagen Burnout Inventory (CBI) for burnout measurement were used.

**Results:**

Medical residents’ perceptions about their educational environment are rather negative. Their job-related stress ranged between moderate and high levels, while burnout ranged in medium levels. A significant positive association was observed between total CBI and its subscales and stress. Positive evaluation of the clinical learning environment was inversely related with burnout levels. Job stress was correlated independently and positively with higher total burnout levels and its’ three dimensions. Work-related burnout was independently and negatively related with social support.

## Introduction

Burnout during residency has gained great attention, while a number of studies have revealed that its’ prevalence among medical residents (MR) is high. According to the results of a literature review that included 51 studies, 27–75% of MR experience burnout [1]. Literature suggest that MR burnout is related with deterioration of general health status, substance abuse, anxiety, depression, suicidal thoughts, poor performance, medical errors and suboptimal patient care [2–5].

A number of personal characteristics (age, personality traits like neuroticism, and low stress tolerance) may lead to the development of the burnout syndrome [6]. However, interpersonal and organizational stressors like workload, time demands, lack of control, work planning and interpersonal relationships are considered important contributing factors in MR burnout [1, 2]. Moreover, the dual role of doctor and a trainee during residency period may set the scene of burnout. Thus, the aspects related to the educational environment and work-related stressors may strongly affect burnout levels of trainee doctors.

Hospital educational environment is of great importance in medical education. An inspiring clinical teaching environment includes good preparation and planning, reflection on learning, good relationship with supervisors and colleagues and successful social integrations for the residents [7, 8]. On the other hand, lack of clear objectives, passive observation, little time for feedback, not focusing on problem-solving skills and teaching by humiliation are some of the most common problems concerning interaction and learning in the clinical environment [7].

Transition to hospital is a difficult and stressful change for most MR [9]. Given the long-hour workload demand on trainee doctors, the demanding patients and environment and the fact that they are both therapists and apprentices, MR are considered to be some of the most vulnerable employees to occupational stress [10]. Their mistakes are highly visible and they are constantly evaluated by patients, relatives, colleagues and supervisors. Consequently, long hours devoted to specialty, many responsibilities, inadequate support from senior staff, job uncertainty and the fact that their actions as doctors have a great impact on human life, are some of the most important stressors [10, 11]. Furthermore, MRs’ dual role is related with contradictory needs and demands, since a trainee’s role is to ask questions, while doctor’s is to answer them.

The aim of the study was to investigate the impact of hospital educational environment and occupational stress on MR burnout. According to our main hypotheses, positive perceptions about the educational environment are negatively related to burnout, while higher work-related stress is positively associated with burnout levels.

## Main text

### Methods

#### Participants and procedure

A cross-sectional study was conducted. Data collection was performed through online questionnaires using Google Docs Forms. All MR personal information were reached through association forums and social media platforms and a link with an anonymous questionnaire was forwarded to them via personal email. From the 382 personal emails distributed, 269 participated in the research.

#### Measures

##### Socio-demographics and information concerning specialty

Research tool contained questions recording both socio-demographic information (sex, age, family status, specialty) and additional information concerning post-graduate studies, hospital of residency, years of internship in total and years of internship in the specific position.

##### Postgraduate Hospital Educational Environment Measure—Greek version (PHEEM-G)

Trainee doctors’ perceptions about their educational environment were assessed by the Greek Version of the Postgraduate Hospital Educational Environment Measure (PHEEM-G) [12]. PHEEM evaluates three domains of the clinical learning environment: (i) perceptions of autonomy; (ii) perceptions of teaching; (iii) and perceptions of social support [13]. Its’ Greek version consists of 40 closed type questions, ranked on a 6-point scale [12]. Additionally, in the PHEEM-G a pre-figuration of all subintervals in the standard scale of 0–100, for both total scales and subscales, has been made through its’ validation process in order to facilitate its understanding and comparison [12]. Following the above adaptation, cut-off scores of the Greek Version are as follows: 0–25 very negative, 26–40 negative, 41–50 rather negative, 51–60 rather positive, 61–75 positive and 76–100 very positive educational environment, for both total PHEEM-G and its’ three facets [12]. Cronbach alpha for the scale was 0.94.

##### Job Stress Measure (JSM)

The Greek version of the Job Stress Measure (JSM-G) was used for the assessment of work related stress [14]. JSM [14, 15] consists of sixteen items rated on a 5-point scale, evaluating stress produced by a number of work related factors such as the amount of responsibility, the volume of work that must be accomplished in an allotted time, the conflict demands that a position presents and the time pressure. A higher score indicates higher stress levels. Cronbach a alpha for the scale was 0.90.

##### Copenhagen Burnout Inventory (CBI)

MR burnout levels were measured with Copenhagen Burnout Inventory (CBI) [16]. The CBI consists of 19 items and evaluates (i) personal (6 items), (ii) work related (7 items) and (iii) client-related (6 items) burnout. Personal exhaustion refers to both physical and psychological fatigue that accumulates in a person during the day, occupational exhaustion describes fatigue that is derived from work, while client-related exhaustion depicts burnout as a consequence of interpersonal relationship with the clients [16]. Higher scores indicate higher burnout levels. Cronbach alpha for the scale was 0.89.

#### Statistical analysis

Scales normality was evaluated by Kolmogorov–Smirnov test. Correlations between variables were examined with Spearman correlation test. Standard multiple regression analysis between JSM-G, PHEEM, and CBI underlined independent relations. Statistical significance was set at 0.05 with a confidence interval of 95%. Analyses were conducted using SPSS (version 24.0).

### Results

The sample included 269 MR. Males were slightly more than females (51.3%), with mean age 31.7 years old (SD = 4.5). Most of them were single (71.0%). Regarding their specialty, 51.3% were trained in a specialty of the internal medicine sector, 41.6% in the surgical sector and a percentage of 7.1% attended a specialty in the laboratory sector. 82.5% attended a full time residency in their current position. Moreover, 39.8% has completed post-graduate studies. Their mean internship duration at the current site was 1.89 years (SD = 1.16) and in general was 3.12 years (SD = 1.68). Socio-demographic features of the sample are presented in Table 1.

**Table 1:** Socio-demographic characteristics of the sample

	Ν	%	Mean	SD
Sex				
Male	138	51.3		
Female	131	48.7		
Age			31.70	4.46
Family status				
Single	191	71.0		
Married	73	27.1		
Divorced	4	1.5		
Widowed	1	0.4		
Sector of specialty				
Internal medicine	138	51.3		
Surgical	112	41.6		
Laboratory	19	7.1		
Type of residency				
Complete	222	82.5		
Partial	47	17.5		
Location of hospital				
Thessaloniki	168	62.5		
Other city	101	37.5		
Post-graduate studies				
MSc	84	31.2		
PhD	23	8.6		
Years of residence			3.12	1.68
Years in current position			1.89	1.16

The results of the descriptive statistics of the three scales are presented in Table 2. According to the pre-figuration of all subintervals in the standard scale of 0–100, for both total scale and subscales of the PHEEM-G [12], MR perceive their educational environment as rather negative in total (Mean = 46.26, SD = 14.54), while at the same time their perception are rather negative for social support, teaching and autonomy. In the case of job stress and burnout there are no specific cut-off scores. However, by taking into account the range of the potential scores (16–80 for JSM, 0–76 for CBI total, 0–24 for personal and patient-related and 0–28 for work-related burnout) we could say that the participants experience moderate stress levels (Mean = 46.97, SD = 11.28). Regarding burnout, means of total CBI (Mean = 31.25, SD = 12.46), personal (Mean = 11.15, SD = 4.35) and work-related (Mean = 12.98, SD = 5.68) exhaustion were at medium levels, while patient-related exhaustion ranged in low levels (Mean = 7.12, SD = 5.03) Table 2.

**Table 2:** Descriptives of CBI, Job Stress Measure and PHEEM tools

	Mean	SD	Range
CBI total	31.25	12.46	0–76
Personal burnout	11.15	4.35	0–24
Work-related	12.98	5.68	0–28
Patient-related	7.12	5.03	0–24
Job stress	46.97	11.28	16–80
PHEEM total	46.26	14.54	0–100
Social support	49.59	14.33	0–100
Teaching	46.80	19.51	0–100
Autonomy	42.09	16.36	0–100

In order to investigate the associations between CBI and its subscales and educational environment and work-related stress a Spearman correlation test was performed (Table 3). A significant positive association was observed between total CBI and its subscales and stress (< 0.001). In all cases, correlation coefficient was considered high (from 0.464 to 0.687), indicating that higher stress is related with higher levels of exhaustion. Total PHEEM scores were significantly and negatively associated with total CBI and its’ subscales, indicating that positive evaluation of the clinical learning environment was inversely related with burnout levels. In all cases except work-related exhaustion (rho = − 0.621) correlation coefficient was considered low (from − 0.124 to − 0.232). Regarding PHEEM subscales social support was negatively related with total CBI and its subscales, while evaluation of autonomy levels and teaching were significantly and negatively associated with total CBI personal and work-related exhaustion (Table 3).

**Table 3:** Correlations between CBI, PHEEM, and Job Stress Measure

	Burnout (CBI)							
	Total		Personal		Work-related		Client-related	
	p	rho	p	rho	p	rho	p	rho
PHEEM								
Social support	< 0.001	− 0.300	0.001	− 0.191	< 0.001	− 0.367	0.003	− 0.177
Autonomy	< 0.001	− 0.287	< 0.001	− 0.241	< 0.001	− 0.355	0.072	− 0.107
Teaching	< 0.001	− 0.232	0.012	− 0.150	< 0.001	− 0.312	0.068	− 0.109
Total score	< 0.001	− 0.232	0.002	− 0.186	< 0.001	− 0.621	0.038	− 0.124
Stress	< 0.001	0.687	< 0.001	0.603	< 0.001	0.621	< 0.001	0.464

Standard multiple regression analysis with CBI scale and subscales as dependent variables and hospital educational environment and stress as covariates followed. Job stress correlated positively and significantly with total CBI score (p < 0.001, B = 0.904, Beta = 0.652). The model explained 49.2% of the variance of exhaustion (R squared = 0.492, p < 0.001). Job stress also correlated independently and positively with higher personal exhaustion (p < 0.001 B = 0.983, Beta = 0.604), explaining 39.4% of its variance (R squared = 0.394 p < 0.001). Work-related burnout was independently and positively related with job stress (p < 0.001 B = 0.879 Beta = 0.562) and negatively with social support subscale of PHEEM (p = 0.048 B = − 0.161 Beta = − 0.132) with this model describing 43.8% of its variance (R squared = 0.438 p < 0.001). Finally, job stress was correlated independently with patient-related burnout (p < 0.001, B = 0.854 Beta = 0.453), explaining 22.6% of its variance (R squared = 0.226 p < 0.001).

### Discussion

The study investigated the impact of hospital educational environment and occupational stress on MR burnout. Residents’ perceptions about their educational environment were rather negative. This is in line with previous studies regarding MR in Greece [17–19]. Dissatisfaction regarding training experience among Greek MR could be attributed to the reasons bellow: first of all, residency programs are not of equal volume, while spectrum of training varies, secondly, rotation of trainees is not legislated, thirdly there are no clear national guidelines for systematic training and, finally, biomedical research is encouraged only in university hospitals [20].

Regarding stress, the participants experienced moderate stress levels, which complies with previous studies in Greece [10, 18]. Besides, findings from studies in other countries confirm the presence of occupational stress among trainee doctors. A research conducted in Germany involving 435 doctors in six different disciplines, showed that occupational stress and depression are frequent occurrences in this category of doctors [11]. Similar were the findings in a study among 1.121 MR in Japan [21] and 433 trainees in Switzerland [22].

In our study, total, personal and work-related burnout were in medium levels, while patient-related exhaustion ranged in low levels. The above finding was contradictory with the results of previous Greek studies. In a research conducted among 311 Greek MR half of the respondents met the burnout criteria [23]. In another study in 263 MR, 14.4% were found to experience burnout [24]. Finally, in a study exploring differences between Greek trainees inside and outside the country, almost one out of three residents in the Greek National Health System (NHS) reported high total burnout levels, compared with the Greek residents in the German and British N.H.S. that demonstrated 5.6% and 3.8% respectively [25].

A significant positive association was observed between total CBI and its subscales and stress, while the model with the work-related stress as an independent variable explained 49.2% of the variance of exhaustion. Although JSM-Gevaluates a number of different aspects, most of the items of the questionnaire are related to workload, which is considered to be the most important stressor leading to higher burnout levels amongst residents. Studies from different countries like Japan, [26] Portugal, [27] Greece [24, 25] and Malaysia [28] revealed similar findings, indicating overwork as the most important predictor for residents burnout. Moreover, a number of studies have shown that the amount of responsibilities and stressors related to career development, that JSM-G also evaluates, are strongly related to residents’ exhaustion [24, 28].

Social support was negatively related with total CBI and its subscales. The inverse relationship between burnout prevalence and collegial support has been mentioned in previous studies [23, 24]. Additionally, autonomy subscale of PHEEM was significantly and negatively associated with total CBI personal and work-related exhaustion. According to the findings of another study, each increased point for autonomy levels was associated with a decrease in the odds of burnout [24].

In summary, exhaustion was positively related with work-related stress and negatively with social support. Future research could focus on preventive factors for burnout development among MR. Since literature suggest that factors within work and learning environment are the most crucial drivers of burnout, rather than individual attributes [29], future interventions may target to stress reduction and improvement of learning environment aspects.

## Limitations

- The study was cross-sectional, so the extent to which the results could be generalized remains limited.
- Participants were selected on the basis of convenience.
- The sample was not geographically representative.

## Data Availability

A confidentiality agreement with participants prevents us from sharing the data.

## Abbreviations

CBI: Copenhagen Burnout Inventory
JSM: Job Stress Measure
MR: medical residents
PHEEM: Postgraduate Hospital Educational Environment Measure
